# Growth Performance and Root Transcriptome Remodeling of Arabidopsis in Response to Mars-Like Levels of Magnesium Sulfate

**DOI:** 10.1371/journal.pone.0012348

**Published:** 2010-08-23

**Authors:** Anne M. Visscher, Anna-Lisa Paul, Matias Kirst, Charles L. Guy, Andrew C. Schuerger, Robert J. Ferl

**Affiliations:** 1 Horticultural Sciences Department, University of Florida, Gainesville, Florida, United States of America; 2 School of Forest Resources and Conservation, University of Florida, Gainesville, Florida, United States of America; 3 Environmental Horticulture Department, University of Florida, Gainesville, Florida, United States of America; 4 Plant Pathology, University of Florida, Kennedy Space Center, Florida, United States of America; 5 Interdisciplinary Center for Biotechnology Research, University of Florida, Gainesville, Florida, United States of America; Iowa State University, United States of America

## Abstract

**Background:**

Martian regolith (unconsolidated surface material) is a potential medium for plant growth in bioregenerative life support systems during manned missions on Mars. However, hydrated magnesium sulfate mineral levels in the regolith of Mars can reach as high as 10 wt%, and would be expected to be highly inhibitory to plant growth.

**Methodology and Principal Findings:**

Disabling ion transporters AtMRS2-10 and AtSULTR1;2, which are plasma membrane localized in peripheral root cells, is not an effective way to confer tolerance to magnesium sulfate soils. Arabidopsis *mrs2-10* and *sel1-10* knockout lines do not mitigate the growth inhibiting impacts of high MgSO_4_·7H_2_O concentrations observed with wildtype plants. A global approach was used to identify novel genes with potential to enhance tolerance to high MgSO_4_·7H_2_O (magnesium sulfate) stress. The early Arabidopsis root transcriptome response to elevated concentrations of magnesium sulfate was characterized in Col-0, and also between Col-0 and the mutant line *cax1-1*, which was confirmed to be relatively tolerant of high levels of MgSO_4_·7H_2_O in soil solution. Differentially expressed genes in Col-0 treated for 45 min. encode enzymes primarily involved in hormone metabolism, transcription factors, calcium-binding proteins, kinases, cell wall related proteins and membrane-based transporters. Over 200 genes encoding transporters were differentially expressed in Col-0 up to 180 min. of exposure, and one of the first down-regulated genes was *CAX1*. The importance of this early response in wildtype Arabidopsis is exemplified in the fact that only four transcripts were differentially expressed between Col-0 and *cax1-1* at 180 min. after initiation of treatment.

**Conclusions/Significance:**

The results provide a solid basis for the understanding of the metabolic response of plants to elevated magnesium sulfate soils; it is the first transcriptome analysis of plants in this environment. The results foster the development of Mars soil-compatible plants by showing that *cax1* mutants exhibit partial tolerance to magnesium sulfate, and by elucidating a small subset (500 vs. >10,000) of candidate genes for mutation or metabolic engineering that will enhance tolerance to magnesium sulfate soils.

## Introduction

Long duration human missions to Mars must rely on more than just stored supplies and physico-chemical means to regenerate air and clean water. The Advanced Life Support (ALS) scenarios envisioned for extended manned missions will depend upon the efficient use of local planetary resources and the recycling of limited materials such as water, pressurized atmosphere, and organic matter, while producing food to augment supplies [1]. The use of in situ regolith for plant growth in a future bioregenerative life support system on Mars may have several advantages over hydroponic systems [2], [3]. These include the immediate bioavailability of plant essential ions, low-tech mechanical support for plants, and easy access to in situ materials once on the surface. However, plant growth may be reduced or inhibited by phytotoxic substances in the regolith, such as high levels of soluble magnesium sulfate minerals. Hydrated forms of magnesium sulfate such as MgSO_4_·7H_2_O (epsomite) and MgSO_4_·H_2_O (kieserite) have been detected in several regions by the Mars Express Satellite [4], [5], [6]. Analyses by the Mars Exploration Rover landers at Meridiani Planum and Gusev crater have also indicated the presence of high levels of magnesium sulfate minerals (up to 10 wt%) in outcrops and soils [7], [8], [9].

High levels of hydrated sulfate minerals in regolith on Mars used in bioregenerative life support systems will lead to exposure of plant roots to supra-optimal concentrations of both Mg^2+^ and SO_4_^2−^ ions in the soil solution. Plants may have evolved to cope with relatively high levels of elements in the soil environment by limiting internal accumulation or tolerating high internal concentrations [10]. In a potential bioregenerative life support system on Mars, an excess of a particular element in the crew's diet could affect the availability of other required elements. This study therefore first determined whether knockout mutant lines for genes encoding certain transporters responsible for uptake of Mg^2+^ and SO_4_^2−^ ions in roots could enhance plant tolerance to high levels of magnesium sulfate in the growth medium and then moved to a molecular analysis of the responses to Mg^2+^ and SO_4_^2−^ in order to increase the potential pool of candidate genes.

Various efforts have previously illustrated that the disabling of transporter genes can indeed improve tolerance to certain elements. For example, a line of transgenic wheat plants expressing an antisense construct of the high affinity K^+^ transporter TaHKT2;1 showed reduced sodium uptake by roots and enhanced growth relative to unstressed plants compared to a control line at high levels of NaCl in the growth medium [11].

Several plasma membrane localized proteins in the outer cell layers of Arabidopsis roots are known to be responsible for magnesium or sulfate ion uptake. Two specific genes, *AtMRS2-10* and *AtSULTR1;2*, were chosen for this study, in part because of their localization and because of availability of knockout lines. *AtMRS2-10* ( = *AtMGT1*) functionally complemented a bacterial mutant lacking Mg^2+^ transport capability and AtMRS2-10-GFP–expressing plants showed fluorescence in the periphery of root cells, suggesting a plasma membrane association [12]. *AtMRS2-10* is, to date, the only member of the *AtMRS2* family that is known to be associated to the plasma membrane of root cells in addition to having Mg^2+^ ion transport capability. Therefore, a knockout mutant line of the *AtMRS2-10* gene (*mrs 2-10*) was characterized for this study based on the SALK_100361.41.30.x T-DNA insertion line [13]. *AtSULTR1;2* is a constitutively expressed sulfate transporter gene whose product AtSULTR1;2 is localized in root hair, epidermal and cortical cells where it ensures sulfate uptake into plants under sulfur-replete conditions [14]. A knockout mutant line of the *AtSULTR1;2* gene (*sel1-10*) was shown to take up 80% less sulfate than wildtype when grown for 12 days on agar with optimal levels (1.7 mM MgSO_4_·7H_2_O) of sulfate [15].

Knockout lines of genes encoding vacuolar H^+^/Ca^2+^ transporters CAX1 and CAX3 were also analyzed to present perspective of other, non-plasma membrane, cellular transporter locations. The *cax1-1* and *cax1/cax3* lines were previously shown to be more tolerant of high Mg^2+^ levels in the form of MgCl_2_ when grown on agar [16], [17]. In a separate study, a *CAX1* knockout mutant was identified through a mutant screen on nutrient solutions reflecting low Ca∶Mg ratios characteristic of serpentine soils [18]. When high MgSO_4_·7H_2_O concentrations are applied, the Ca∶Mg ratio of the nutrient solution decreases, which therefore makes the *cax1-1* and *cax1/cax3* mutants candidates for high magnesium sulfate tolerance. This study determined whether enhanced tolerance to low Ca∶Mg ratios is maintained when *cax1-1* and *cax1/cax3* mutants are exposed to elevated SO_4_^2−^ in addition to elevated Mg^2+^ in soil medium.

These candidate genes for enhanced magnesium sulfate tolerance that are presented above were selected based on previous literature describing the results of individual gene or gene family analyses. Genome-wide transcriptome analyses of plants exposed to elevated concentrations of magnesium, sulfate, or magnesium sulfate in the growth medium have not been reported to date. In order to increase the number of candidate genes that could play a role in enhancing growth performance of plants exposed to this abiotic stress, root transcriptome remodeling in Col-0 was analyzed after short-term (45 min.) hydroponic exposure to a non-lethal, high concentration of MgSO_4_·7H_2_O. This early time point was chosen to capture part of the primary stress responses. The differential expression of transporter genes was analyzed after 90 min. and 180 min. in addition to 45 min., as this group in particular may represent a metabolic strategy for tolerance to an elevated magnesium sulfate environment. The root transcriptome of the transporter gene knockout mutant *cax1-1* was furthermore contrasted with that of Col-0 after both genotypes were exposed to MgSO_4_·7H_2_O treatment for 180 min. Based on their previously identified tolerance to high MgCl_2_ and low Ca∶Mg ratios, *cax1* mutants are likely to show enhanced growth compared to wildtype when exposed to high magnesium sulfate levels. Furthermore, ionome analyses of *cax1* mutants showed that they have significantly reduced levels of Mg^2+^ in their leaves compared to wildtype [18].The *cax1/cax3* double knockout mutant, while having the potential to show tolerance to elevated MgSO_4_·7H_2_O based on its tolerance to MgCl_2_ and its reduced shoot levels of Mg^2+^, showed significant differences in concentration of multiple elements, and grew more slowly than wildtype under regular nutrient conditions [17]. Since *cax1-1* mutants did not display negative effects on growth under regular nutrient conditions, *cax1-1* rather than *cax1/cax3* was included in the root transcriptome analysis. Genes differentially regulated between *cax1-1* and Col-0 grown under stress conditions could reveal those molecular processes possibly leading to significant differences in growth performance at the whole plant level. A hydroponic medium was chosen to mimic root exposure to a specific soil solution in order to directly link transcriptome responses to the level of bioavailable ions that roots may experience in a hypothetical root zone of a regolith profile on Mars [19]. The treatment concentration of MgSO_4_·7H_2_O was based on the low Ca∶Mg ratio that can occur in serpentine soils on Earth. Because of their high amount of bioavailable magnesium, Serpentine soils can be seen as a partial analogue for regolith high in magnesium sulfate on Mars [20].

## Materials and Methods

### *Mrs2-10* T-DNA Insertion Line Characterization

**Figure 1:**
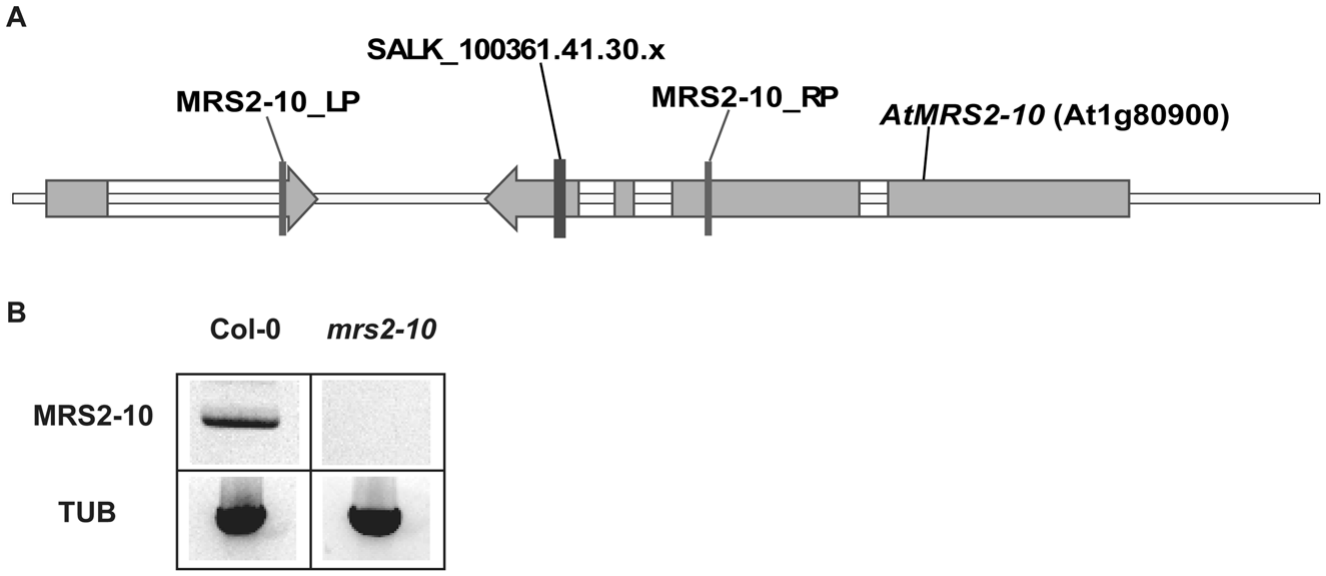
Homozygous knockout mutant line *mrs2-10*. (a) A homozygous T-DNA insertion mutant for *AtMRS2-10* (At1g80900) was identified by PCR based on the SALK_100361.41.30.x line. (b) RNA extracted from *mrs2-10* and Col-0 leaves was tested for the presence of *MRS2-10* mRNA with gene-specific primers. Alpha-(α)-Tubulin (TUB) was used as a constitutive control.

A homozygous knockout T-DNA insertion line for *AtMRS2-10* (At1g80900) was identified by PCR and RT-PCR using the original SALK_100361.41.30.x line (Fig. 1a,b). For PCR, oligonucleotide primers MRS2-10_LP (5′-CAGGATCAAAGCATCGTTCTC-3′) and MRS2-10_RP (5′-TAGGAGCTCAGAAGACGCAAC-3′) were designed using software available on the Salk Institute website (http://signal.salk.edu/tdnaprimers.html). In addition, the T-DNA specific primer LBb1 (5′-GCGTGGACCGCTTGCTGCAACT-3′) was included as designed and recommended by the Salk Institute. Combinations of the primers were used to identify plants for which the T-DNA insertion was present in both AtMRS2-10 alleles. Genomic DNA was extracted from T-DNA insertion mutant leaves using the Shorty method. For this method, 500 uL Shorty Buffer (0.2 M Tris-HCL pH 9.0, 0.4 M LiCl, 25 mM EDTA, and 1% SDS) was added to ground leaf tissue (∼0.25 cm^2^) in a microcentrifuge tube. The tube was then spun for 5 min. at high speed in a microcentrifuge. Supernatant (350 uL) was transferred to a new tube containing isopropanol (350 uL). The two volumes were mixed by inversion and the tube was spun for 10 min. at top speed in a microcentrifuge. After this, the liquid was discarded and the pellet was air-dried before being resuspended in 100 uL TE buffer. Genomic DNA was extracted from Col-0 leaves using the DNeasy Plant Mini Kit (Qiagen). PCR was carried out using JumpStart Taq DNA polymerase (Sigma). PCR products were separated in agarose gels and stained with SYBR Safe DNA gel stain (Invitrogen). A confirmed homozygous T-DNA insertion mutant was backcrossed to Col-0 three times before allowing self-fertilization. Homozygous plants backcrossed 3× were identified by PCR and used for seed generation. For RT-PCR, RNA was extracted from leaves of Col-0 and a homozygous T-DNA insertion line for *AtMRS2-10* (At80900) with the RNeasy Plant Mini Kit (Qiagen). Gene specific oligonucleotide primers MRS2-10_LP1 (5′-AGGGTTACTTTGTCGGAGA-3′) and MRS2-10_RP1 (5′-TACACGGGGTTTTATCTTG-3′) were designed based on Arabidopsis genomic DNA sequence information (NCBI). Alpha-(α)-Tubulin was used as a constitutive control with primers according to Yoshimoto (2002). RT-PCR was carried out using the OneStep RT-PCR kit (Qiagen). PCR products were separated in agarose gels and stained with SYBR Safe DNA gel stain (Invitrogen). An ionome analysis of the backcrossed homozygous *AtMRS2-10* knockout mutants (*mrs2-10*) was performed as part of the Purdue Ionomics project and results are available online through the Purdue Ionomics Information Management System (http://www.ionomicshub.org/home/PiiMS) [21].

### Plant Growth Experiments on Soil Medium

Seeds from the Ws, Col-0, *mrs2-10*, *sel1-10* [15], *cax1-1* [16] and *cax1/cax3* [17] lines were sterilized by soaking them for 15 minutes in a 40% (v/v) bleach solution with a drop of Tween 20 (polyoxyethylenesorbitanmonolaureate). The seeds were then washed six times with sterilized (autoclaved) water. To control for environmental variation when comparing plant growth per concentration, each tray was divided in 8 sections of 6 wells each, with either a mutant line or its associated wildtype background line planted in one section, in alternating fashion. This method resulted in 4 sections per seed line per tray. The seeds were evenly planted (5 seeds per tray well) on the soil medium (sphagnum peat moss, vermiculite and perlite) that was wetted with nutrient solution. The basic and control nutrient solution consisted of 2.2 g/L Murashige and Skoog salts (Sigma-Aldrich) and 0.5 g/L MES buffer (Sigma-Aldrich). For the treatments, MgSO_4_·7H_2_O (Sigma-Aldrich) was added to the basic solution at 20, 60, 80 or 100 mM concentration. The pH of the solutions was adjusted to 5.70–5.75 with KOH. The trays with soil were covered with plastic wrap to maintain sufficient humidity and placed in a cold room at 4°C for three days. After stratification, the trays were moved to a growth room at 23°C where they were randomly placed on the growth benches. There, the seeds germinated, the plastic wrap was removed after 3–5 days, and the surviving plants were thinned to two plants per well (maximum of 12 plants per section) after one week to maintain sufficient spacing. The plants were sub-irrigated twice a week, alternatively with tap water and the appropriate nutrient solution. The plants were analyzed at week 4 and the experiment was repeated three times.

### Growth Performance Measurements and Statistical Analysis

Fresh weight biomass of plants grown on soil was measured by grouping and weighing whole shoots of plants per tray section. Leaf chlorophyll content of plants grown on soil medium was measured with a Minolta SPAD-502 meter. One leaf measurement per plant per tray well was taken. Biomass or chlorophyll levels of wildtype and knockout mutant Arabidopsis lines were evaluated in a mixed analysis of variance (ANOVA) model using SAS 9.2 and JMP Genomics 7 software. Genotype and concentration were included in the ANOVA model as fixed effects, while batch (replication set) was included as a random effect. Unbiased estimates of biomass or chlorophyll levels (least-square means) were generated for each effect (genotype, concentration, or genotype and concentration) per experiment. The estimated levels of biomass or chlorophyll were then compared using a series of t-tests, generating estimated differences and corresponding p-values. Differences with associated p-values<0.05 were considered significant. Results of the ANOVA analysis are presented in Table S1.

### Hydroponic Root Growth

**Figure 2:**
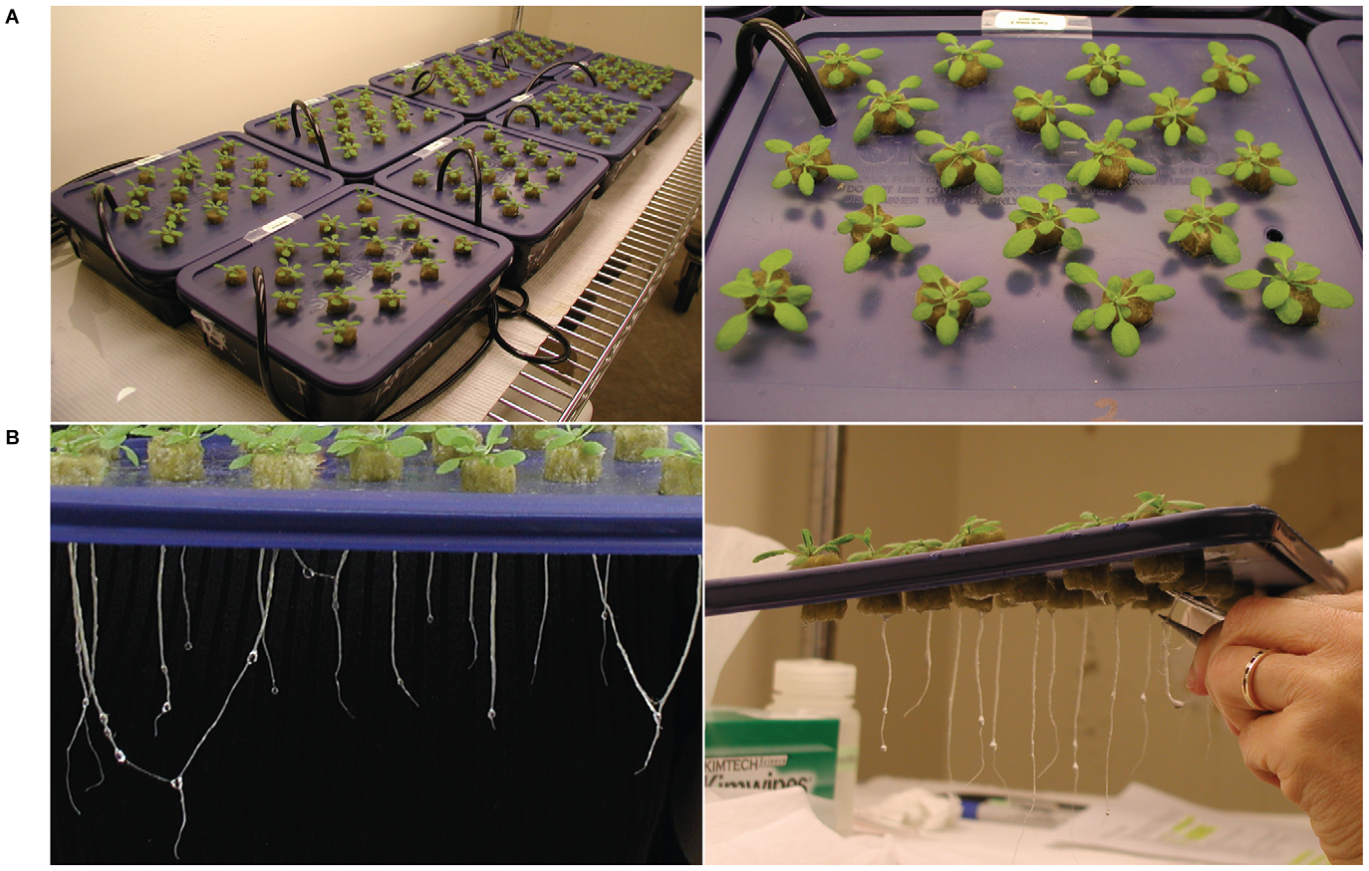
Overview of hydroponic Arabidopsis growth and root harvest. (a) Set-up for the microarray experiment. (b) Example of Arabidopsis roots after 21 days of growth, and of root harvest per container for the microarray and Q-PCR experiments.

The hydroponic set-up for Arabidopsis that was used in this study is based on two previously described systems [22], [23]. Glass containers (2.6 L) were covered with opaque black plastic sheeting along the glass, and with opaque plastic lids across the top. In each opaque plastic lid, 18 holes (d = 1.43 cm) were made at regular intervals with a hole puncher. A 2-cm-long Rockwool (Grodan) plug (d = 1.59 cm) was placed in each hole. Containers were filled with a nutrient solution adjusted to pH 5.7 by KOH that was replaced once a week. It consisted of distilled water, 0.25 g/L MES buffer, and 1/32x MS salts during week 1 and 2, while the concentration of MS salts increased to 1/16x during week 3. Arabidopsis seeds were sterilized and stratified before planting about ten seeds per Rockwool plug. Col-0 seeds were planted on 18 containers while *cax1-1* seeds were planted on 6. Containers were covered with transparent plastic wrap during the first 3–5 days of germination. Holes were made in the plastic wrap after 1–3 days depending on the general atmospheric humidity. The containers were randomly placed over 3 benches (8 containers/bench). Air was supplied to the roots by one 3W air pump per 8 containers; each container had an airstone made from glass beads. After one week, seedlings were thinned to one seedling per Rockwool plug, so that every container supported 18 plants (Fig. 2a). At day 21, Col-0 and *cax1-1* roots were exposed to the basic nutrient solution (0.25 g/L MES, 1/16x MS, pH 5.7) with an additional 2.08 mM magnesium sulfate (total Ca∶Mg ratio = 1∶15). Col-0 was exposed for 45 min., 90 min., or 180 min., while *cax1-1* was exposed for 180 min. only. The Col-0 control set received no extra magnesium sulfate and was harvested at 45 min. together with the first Col-0 treatment set. Four replicate containers were harvested for the control and each of the treatment sets (Fig. 2b). Roots were cut below the Rockwool plug and pooled per container before being flash frozen in liquid nitrogen and stored at −80°C.

### Microarray Procedures, Statistical Analysis and Data display

**Figure 3:**
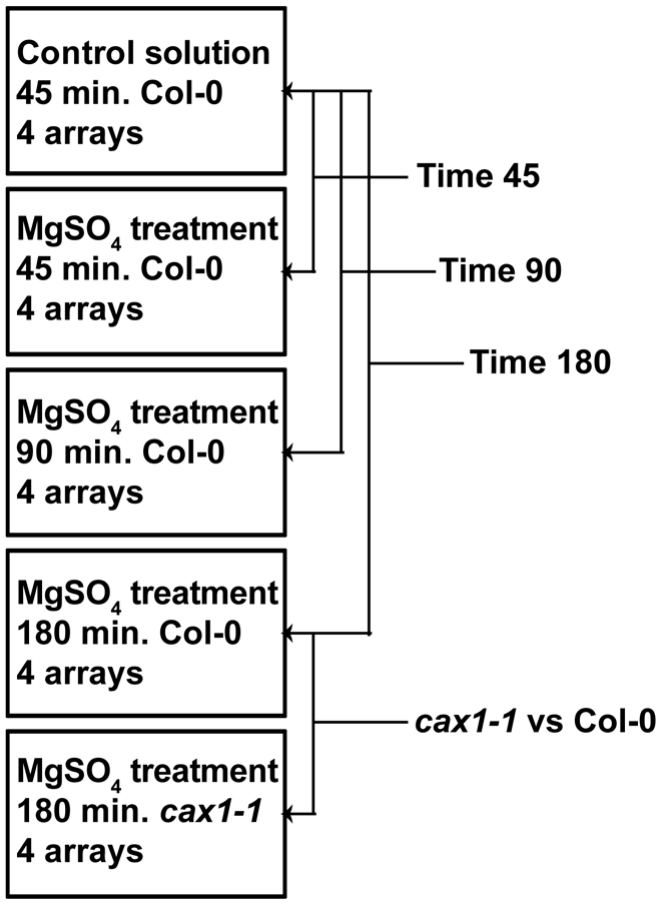
Overview of the microarray experiment. The experiment included 20 arrays in total, each with a single cy3-labeled sample hybridized to it. Each sample set consisted of 4 biological replicates. Gene expression was compared among the sets as indicated by the arrows; Col-0 sets treated for 45 min., 90 min., or 180 min. were compared to the Col-0 set exposed to a control solution for 45 min., and these three comparisons are referred to in the text, tables and figures as Time 45, Time 90, and Time 180 respectively. The comparison of *cax1-1* with Col-0 treated for 180 min. is not referred to in abbreviated form.

RNA was extracted from root samples with the RNeasy Plant Mini Kit (Qiagen). Samples were weighed while still frozen and reagents were adjusted to the total measured weight for the grinding and lysing step. Two replicate extractions were completed per sample with lysate volumes corresponding to 100 mg frozen wet weight each. The remainder of a sample was stored at −80°C as cleared lysate according to the Qiagen protocol. The quality and quantity of the extracted RNA was checked with denaturing agarose gels stained with ethidium bromide and the NanoDrop 1000 Spectrophotometer (Thermo Scientific). RNA from each sample was amplified and labeled with cy3 dye by using Agilent Quick Amp labeling kit, one color. A total of 20 samples were hybridized to five 4×44k Arabidopsis microarray slides (Agilent), which were then washed and scanned before data was extracted. Microarray handling and data extraction was done at the Interdisciplinary Center for Biotechnology Research (ICBR) at the University of Florida according to the One-Color Microarray-Based Gene Expression Analysis (Quick Amp Labeling) Protocol (version 5.7). Median signal intensities were quantile normalized using R software. Log2 transformed normalized data were evaluated using a mixed analysis of variance (ANOVA) model [24], [25] implemented in SAS 9.2 that included the fixed effect of each genotype and time of tissue harvest. Unbiased, least square means estimates of transcript abundance were generated and gene expression measured in each treatment was then compared to a control using a series of t-tests; Col-0 sets treated for 45 min., 90 min., or 180 min were compared to the Col-0 set exposed to a control solution for 45 min., and these three comparisons are referred to in the text, tables and figures as Time 45, Time 90, and Time 180 respectively (Fig. 3). In addition, the *cax1-1* set treated for 180 min. was compared to the Col-0 set treated for 180 min. (Fig. 3). The p-values generated in these comparisons were corrected for multiple testing by controlling the False Discovery Rate (FDR) with the Q-value procedure in the Q-value 1.0 package (default settings) of the R software [26]. A default q-value threshold of 0.05 was used to declare a significant difference in gene expression between treatment and control. The results of the ANOVA analysis are presented in Table S2. Lists of genes with statistically significant changes in expression were further organized, analyzed and displayed in tables and figures using JMP Genomics 7, R software, GeneVenn, GOstat, Cluster 3.0, Java TreeView 1.1.4r5, the National Center for Biotechnology Information (NCBI) BLAST tool, and the Arabidopsis Information Resource (TAIR) Motif analysis tool.

### Quantitative Real-time PCR

Wildtype Arabidopsis plants (Col-0) were grown hydroponically for 21 days as described above. At day 21, four containers received a replacement of the basic nutrient solution (0.25 g/L MES, 1/16x MS, pH5.7), while another four containers received the basic solution with an additional 2.08 mM magnesium sulfate (total Ca∶Mg ratio = 1∶15). Roots of treatment and control plants were exposed for 180 min. before being harvested per container. RNA was extracted and checked for quality and quantity as described above. RNA from these independently grown plants exposed or unexposed for 180 min., as well as RNA from plants exposed for 180 min. and unexposed for 45 min. in the microarray experiment, were used for the quantitative PCR (Q-PCR) analyses. Q-PCR was performed using TaqMan reverse transcription reagents and Power SYBR Green PCR Master Mix (Applied Biosystems). 1 ug of total RNA was reverse transcribed per sample for a total of three control and three treatment samples. Primers were designed for 5 genes using Primer Express (Applied Biosystems) (Table 1). At2g32170 was chosen as a stably expressing reference gene appropriate for abiotic stress treatment [27]. Each template/primer pair combination was run in triplicate. The relative increase or decrease of mRNA abundance between the two sample sets was calculated by using the Pfaffl method, and statistical analysis of the results was done with the REST 2008 2.0.7 software.

**Table 1:** Genes and related primer sequences selected for Q-PCR analysis.

Gene ID	Primer sequence
At2g32170 (reference gene)	FW: 5′-GTTAAATCATGACCATGGCAGTGT-3′
	RV: 5′-CTACATCAACCAGAGGAACATGTGT-3′
At2g38170 (*CAX1*)	FW: 5′-GCGACTCAGATTGGCTTATTCG-3′
	RV: 5′-GATCCATATTAATTCCCAAAATCCA-3′
At1g80900 (*MRS2-10*)	FW: 5′-TTCTCTGTCTGCGCCAGTTTC-3′
	RV: 5′-GGCTCCTTACAATGCTCAAGCT-3′
At3g15990 (*SULTR3;4*)	FW: 5′-GGTGAAGCTGTGGCTGATCTC-3′
	RV: 5′-GCTCCATCTTCAGAAACAGTCTCTCT-3′
At1g80830 (*NRAMP1*)	FW: 5′-ACAGGATCTGGACGGTCTCAA-3′
	RV: 5′-GATGAGTGGAGAATTGGAGAAGCT-3′

## Results

### Plant Growth Performance

**Figure 4:**
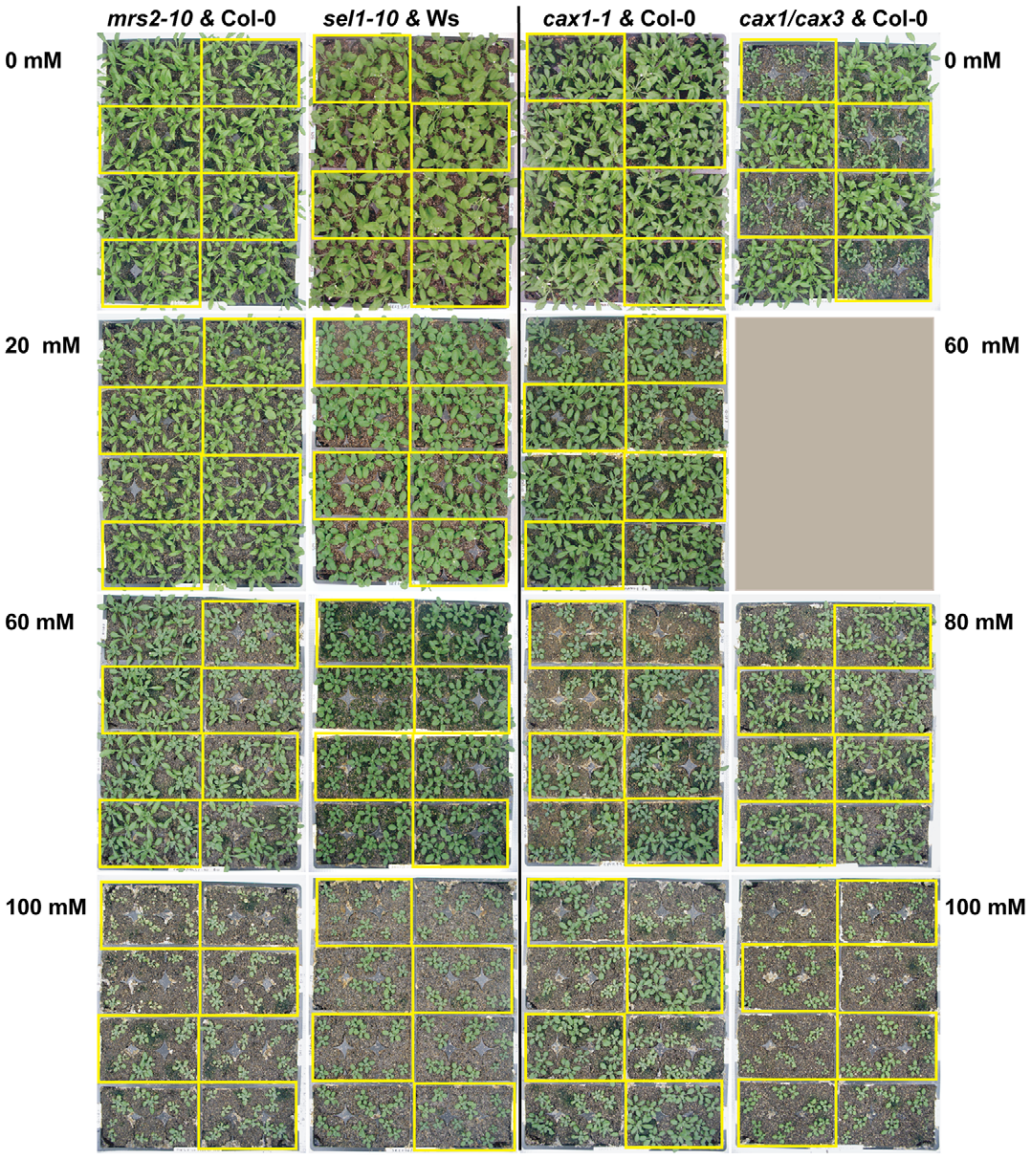
Overview of the Arabidopsis growth experiment on soil. Mutant lines are grown alongside their respective wildtype backgrounds on trays containing soil medium with different levels of dissolved MgSO_4_·7H_2_O. To control for environmental variation, each tray was divided in 8 sections, with a mutant line and its associated wildtype background line planted in alternating fashion. Yellow boxes highlight the sections with mutant plants within each flat; the other sections contain wildtype plants. For the comparison between *cax1/cax3* and Col-0, 60 mM was not tested. The plants shown here are 3 weeks old and were harvested and analyzed after completing 4 weeks of growth. The experiment was repeated three times.

**Figure 5:**
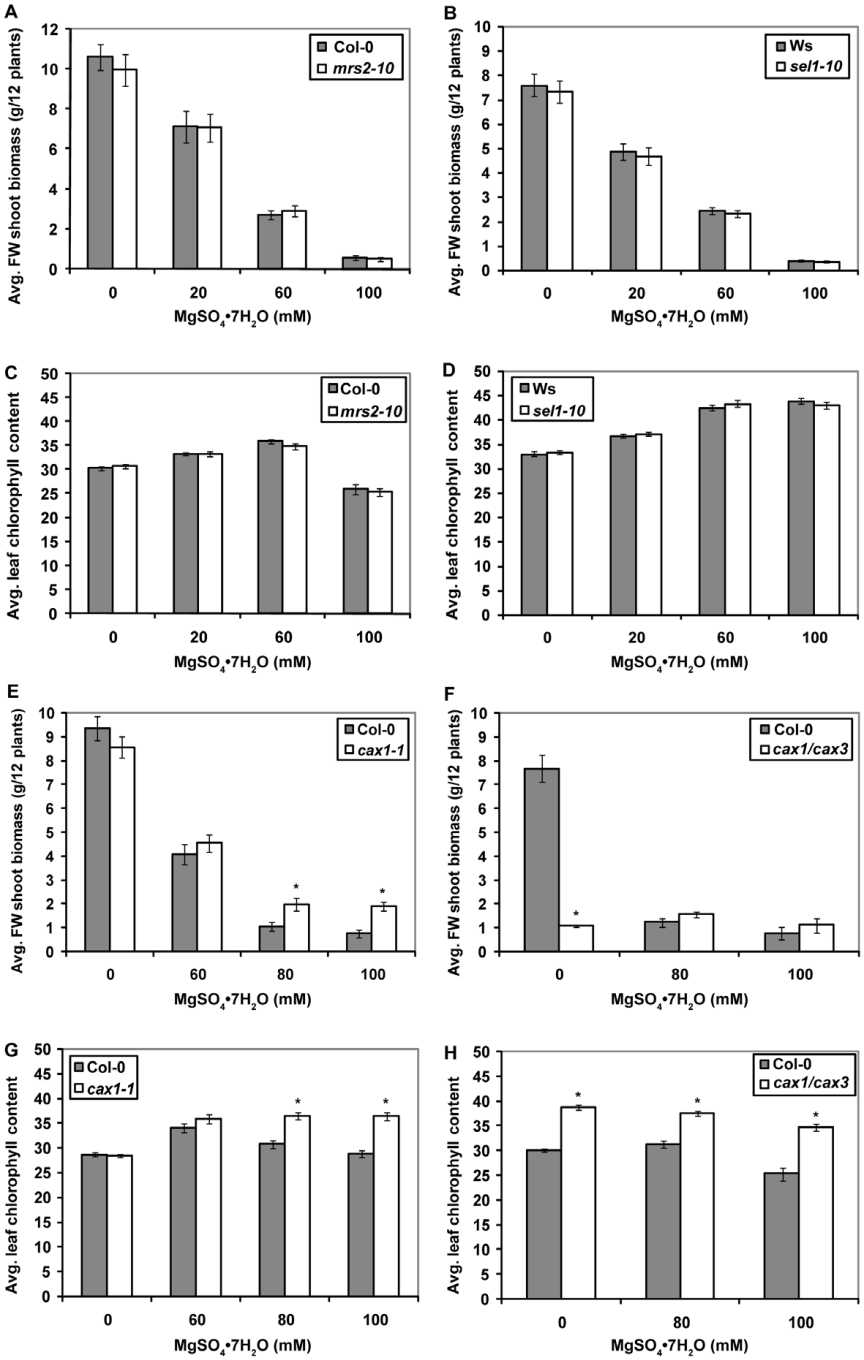
The shoot fresh weight biomass comparisons of mutant and wildtype lines grown on soil. Average fresh weight shoot biomass of (a) *mrs2-10* and Col-0, (b) *sel1-10* and Ws, (e) *cax1-1* and Col-0, or (f) *cax1/cax3* and Col-0 plants in response to increasing concentrations of MgSO_4_·7H_2_O in soil medium. Bars indicate standard error, n = 12. Average leaf chlorophyll content of (c) *mrs2-10* and Col-0, (d) *sel1-10* and Ws, (g) *cax1-1* and Col-0, or (h) *cax1/cax3* and Col-0 plants in response to increasing concentrations of MgSO_4_·7H_2_O in soil medium. Bars indicate standard error, n = 72. The asterisks indicate statistically significant differences between genotypes (p<0.05) at specific concentrations of MgSO_4_·7H_2_O based on ANOVA.

The growth performance of transporter gene knockout mutant lines and their respective wildtype backgrounds grown for four weeks on soil treated with MgSO_4_·7H_2_O in solution (0–100 mM) was compared on the basis of shoot fresh weight (FW) biomass and leaf chlorophyll levels. Figure 4 shows the growth habit of *mrs2-10* and *sel1-10* plants compared to wildtype in the left hand panels, and of *cax1-1* and *cax1/cax3* compared to wildtype in the right-hand panels. Statistical analysis of the FW shoot biomass of wildtype Arabidopsis (Ws, Col-0) with ANOVA confirmed the significant phytotoxic effects of increasing concentrations of dissolved MgSO_4_·7H_2_O on wildtype plant growth (Table S1). Subsequent ANOVA analyses assessed whether selected mutant lines could alleviate the growth-limiting effects seen in wildtype. Results showed that FW shoot biomass and leaf chlorophyll content of *mrs2-10* and *sel1-10* lines were indistinguishable from that of wildtype at the tested concentrations (Fig. 5a–d, Table S1). Ionome analysis of the *mrs2-10* mutant exposed to regular nutrient conditions furthermore did not reveal a statistically different leaf Mg content compared to Col-0 (http://www.ionomicshub.org/home/PiiMS). Although there was little difference between the *mrs2-10* and *sel1-10* lines and their respective wild-type backgrounds, the *cax1-1* and *cax1/cax3* lines showed relative improvement in their ability to grow on MgSO_4_·7H_2_O enriched soil. *Cax1-1* plants had significantly higher FW shoot biomass and leaf chlorophyll content than Col-0 grown at 80 and 100 mM MgSO_4_·7H_2_O (Fig. 5e,g, Table S1). The increase in *cax1-1* shoot biomass over that of Col-0 was 89% and 149% at 80 and 100 mM respectively. The CAX1 knockout mutation did not fully eliminate the effects of high magnesium sulfate on plant growth performance; the absolute FW shoot biomass of *cax1-1* grown on soil for four weeks was still low (20%) compared to untreated Col-0 (Fig 5e). *Cax1/cax3* plants showed significantly higher leaf chlorophyll content than Col-0 grown at 80, and 100 mM, but the average FW shoot biomass increases of 26.8% and 33.2%, at 80 and 100 mM, were not found to be statistically significant (Fig. 5f,h, Table S1). The significant differences in FW shoot biomass and leaf chlorophyll content between *cax1/cax3* and Col-0 grown at 0 mM MgSO_4_·7H_2_O were described in detail by Cheng et al. (2005). In this study, 0 mM was only included as a point of reference for the high concentrations of MgSO_4_·7H_2_O.

### Root Transcriptome

**Figure 6:**
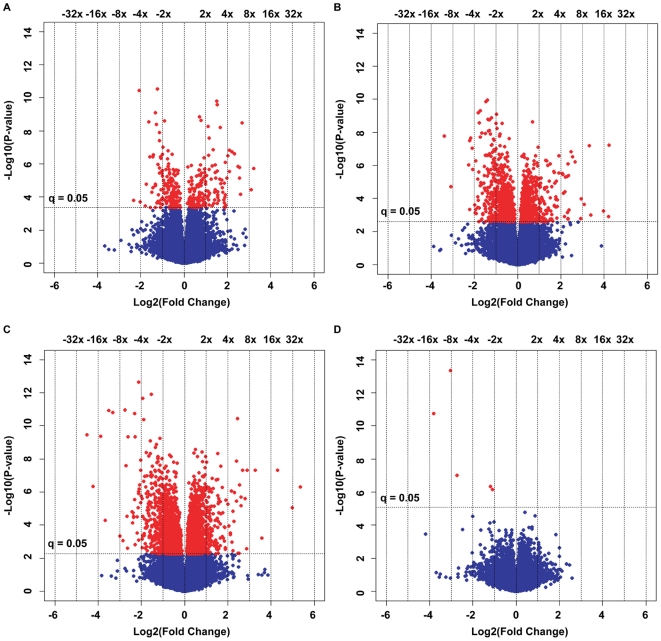
Volcano plots of gene expression comparisons at distinct timepoints. (a) Time 45 (Col-0 treated for 45 min. vs Col-0 exposed to a control solution for 45 min.) (b) Time 90 (Col-0 treated for 90 min. vs Col-0 exposed to a control solution for 45 min.) (c) Time 180 (Col-0 treated for 180 min. vs Col-0 exposed to a control solution for 45 min.) (d) *cax1-1* treated for 180 min. vs Col-0 treated for 180 min. The x-axes show log2 values of the fold changes in gene expression between sample sets. Each dot represents one of 37478 transcripts. Vertical lines indicate absolute fold change values as indicated on top of the graphs. The y-axes show the −log10 p-values corresponding to the log2 fold change values. The horizontal line indicates the −log10 p-value where the q-value is 0.05. Transcripts whose expression difference (fold change) corresponds to a p-value for which q<0.05 are above the horizontal line and indicated in red.

Root transcriptome remodeling was analyzed in Arabidopsis after hydroponic exposure to a non-lethal, high concentration of MgSO_4_·7H_2_O (Fig. 2, Fig. 3, Table S2). The number of genes with statistically significant differences in expression between Col-0 exposed to MgSO_4_·7H_2_O and Col-0 exposed to control conditions increased from 325 (Time 45) to 1516 (Time 90) and 3265 (Time 180) (Fig. 6). The number of genes with a significant difference in expression of over 2-fold increased accordingly, from 100 to 248 and 445. Between *cax1-1* and Col-0 exposed to MgSO_4_·7H_2_O for 180 min., only 4 unique transcripts showed a significant difference in expression, and all were over a 2-fold change in abundance (Fig. 6).

### Primary MgSO_4_·7H_2_O stress response

The primary root transcriptome responses to MgSO_4_·7H_2_O stress were analyzed in Col-0 treated for 45 min. versus Col-0 treated with a control solution for 45 min. (Time 45) to reveal genes involved in the metabolic response to this environment as candidate genes that could play a role in high magnesium sulfate tolerance. The resulting set of 325 genes with significant differences in expression was analyzed for over- and under-represented GO molecular function categories. Significantly over-represented categories were polygalacturonase activity, calcium ion binding, and transcription factor activity (Table 2). Many of the transcripts represented at Time 45 encode phytohormone associated proteins, transcription factors, Ca^2+^-binding proteins, kinases, phosphatases, disease resistance proteins, cell wall related proteins, membrane-based transporters, as well as proteins of unknown functional category. The better characterized of these genes are listed by category in Table 3. For example, phytohormone associated genes encoding enzymes involved in ABA (9-*cis*-epoxycarotenoid dioxygenase), ethylene (1-aminocyclopropane-1-carboxylate synthase) and jasmonic acid (lipoxygenase ) biosynthesis pathways show up-regulated expression. The gene encoding C2H2 type zinc finger family transcription factor ZAT7 is up-regulated, while the gene encoding transcription factor WRKY70 is down-regulated at Time 45. The expression of the gene encoding CBL-interacting protein kinase CIPK9 is up-regulated. Genes encoding cell-wall and disease-resistance associated proteins are also well represented; fasciclin-like arabinogalactan proteins, UDP-glucose 4-epimerase (RHD1), xyloglucan endotransglucosylase (XTH9) and beta-expansin (EXPB1) show down-regulated expression, while the expression of several genes encoding alpha-expansin proteins is up-regulated at Time 45. Well-characterized genes encoding membrane based transporters that are differentially expressed include those encoding calcium exchanger CAX1, calcium-transporting ATPase ACA2, cyclic nucleotide-gated channels CNGC19 and CNGC1, potassium transporter HAK5 and inorganic phosphate transporter PHT1. The 500 bp upstream regions of the down-regulated and up-regulated genes at Time 45 were statistically analyzed for over-represented six-mer sequences (motifs), which may correspond to cis-regulatory elements. Table 4 lists the ten motifs with the highest significance (lowest p-values) for both the up-regulated and down-regulated subsets of genes. The motif with the highest significance in the subset of up-regulated genes is an ABRE coupling element (ABRE-CE). The 325 differentially expressed genes at Time 45 were furthermore compared with a cluster of 197 genes that are known to be differentially expressed in response to a broad range of stress conditions, including cold, osmotic stress, salinity, wounding, and biotic stresses [28]. The comparison showed that at least 18 of the 325 genes differentially expressed at Time 45 appear to be universally responsive to stress conditions (Table 5). These include for example genes encoding Ca^2+^-binding, kinase, zinc finger, and disease resistance proteins.

**Table 2:** Significantly over-represented GO molecular function categories within the set of genes with significant differences in expression at Time 45.

GO category	Molecular function	Count	P-Value
**Over-represented**			
GO:0004650	Polygalacturonase activity	6	0.0176
GO:0005509	Calcium ion binding	8	0.0176
GO:0003700	Transcription factor activity	27	0.0443

The set of 325 genes with significant differences in expression at Time 45 was analyzed for over- and under-represented GO molecular function categories. The results were corrected for multiple testing using False Discovery Rate and the p-value threshold was set to 0.05.

**Table 3:** Well-characterized genes with significant differences in expression at Time 45 (Col-0 treated for 45 min. vs Col-0 exposed to a control solution for 45 min.).

**Transporters**:		
Gene	Description	Log2
At2g38170	calcium exchanger (CAX1)	−0.5676
At4g13420	potassium transporter (HAK5)	1.5548
At5g43350	inorganic phosphate transporter (PHT1) (PT1)	0.3541
At4g37640	calcium-transporting ATPase 2 (ACA2) PM-type	0.3525
At3g17690	cyclic nucleotide-binding transporter 2 (CNGC19)	0.9209
At5g53130	cyclic nucleotide-regulated ion channel (CNGC1)	−0.3754
**Transcription factors:**		
Gene name	Description	Log2
At3g46090	zinc finger (C2H2 type) family protein (ZAT7)	0.9947
At3g56400	WRKY family transcription factor (WRKY 70)	−0.9454
**Kinases:**		
Gene name	Description	Log2
At1g01140	CBL-interacting protein kinase 9 (CIPK9)	1.7794
**phytohormone associated:**		
Gene name	Description	Log2
At1g78390	9-cis-epoxycarotenoid dioxygenase	2.554
At5g65800	1-aminocyclopropane-1-carboxylate synthase	1.8605
At1g72520	lipoxygenase	1.3473
**Cell wall related:**		
Gene name	Description	Log2
At2g23130	arabinogalactan-protein (AGP17)	−1.149
At2g23130	arabinogalactan-protein (AGP17)	−0.7107
At1g55330	arabinogalactan-protein (AGP21)	−1.0069
At5g10430	arabinogalactan-protein (AGP4)	−0.4932
At5g65390	arabinogalactan-protein (AGP7)	−0.7772
At4g12730	fasciclin-like arabinogalactan-protein (FLA2)	−0.3949
At1g03870	fasciclin-like arabinogalactan-protein (FLA9)	−0.501
At1g64440	UDP-glucose 4-epimerase (RHD1)	−0.5767
At4g03210	xyloglucan endotransglycosylase (XTH9)	−0.4144
At2g20750	beta-expansin (EXPB1)	−1.0351
At1g20190	alpha-expansin (EXP11)	0.7098
At3g15370	alpha-expansin (EXP12)	3.2099
At4g01630	alpha-expansin (EXP17)	2.4955
At5g02260	alpha-expansin (EXP9)	0.793

**Table 4:** Motif analysis of 500bp upstream region of genes with significant differences in expression at Time 45 (Col-0 treated for 45 min. vs Col-0 exposed to a control solution for 45 min.).

oligomer	motif name	query set	genomic set	p-value
**up-regulated genes**				
ACGCGG/CCGCGT	ABRE-CE	33/156	1155/33518	5.11E-17
CGCGTA/CGCGTA		28/156	1163/33518	9.30E-13
AACGCG/CGCGTT		32/156	1731/33518	1.72E-11
ACGCGT		24/156	1053/33518	1.36E-10
ACACGG/CCGTGT		31/156	1927/33518	1.04E-09
CGCGTC/GACGCG		23/156	1148/33518	3.74E-09
CACGCG/CGCGTG	D-box	26/156	1525/33518	8.63E-09
AACACG/CGTGTT		48/156	4731/33518	5.07E-08
ACACGT/ACGTGT		51/156	5594/33518	4.64E-07
ACCGCG/CGCGGT		17/156	940/33518	1.72E-06
**down-regulated genes**				
ACAGCT/AGCTGT		40/153	3998/33518	7.44E-07
AATAGA/TCTATT		40/153	13809/33518	3.79E-05
GACAGC/GCTGTC		23/153	2068/33518	4.57E-05
AATATG/CATATT		87/153	13955/33518	5.03E-05
AGCCTG/CAGGCT		20/153	1727/33518	8.48E-05
AATAAT/ATTATT		120/153	21856/33518	1.33E-04
ATATTA/TAATAT		106/153	18637/33518	1.72E-04
ATTAGC/GCTAAT		47/153	6439/33518	2.17E-04
CAGCTG		16/153	1317/33518	2.54E-04
AAGACA/TGTCTT		72/153	11483/33518	3.17E-04

The 500 bp upstream regions of the significantly down-regulated and up-regulated genes at Time 45 were statistically analyzed for over-represented six-mer sequences (motifs), which may correspond to cis-regulatory elements. Table 4 lists the ten motifs with the highest significance for both the up-regulated and down-regulated subsets of genes.

**Table 5:** Genes with significant expression differences between Col-0 treated for 45 min. and Col-0 exposed to a control solution for 45 min.

Gene	Description	Log2
At1g02400	gibberellin 2-oxidase, putative	0.6555
At1g18740	expressed protein	0.706
At1g19180	expressed protein	0.8927
At1g73540	MutT/nudix family protein	0.7802
At1g74450	expressed protein	1.1068
At2g27080	harpin-induced protein-related	0.5201
At2g30040	protein kinase family protein	0.9232
At2g34930	disease resistance family protein	1.3017
At2g41410	calmodulin, putative	−0.78
At2g46600	calcium-binding protein, putative	−1.0632
At3g10300	calcium-binding EF hand family protein	1.1562
At3g16720	zinc finger (C3HC4-type RING finger) family protein	−0.4137
At4g24570	mitochondrial substrate carrier family protein	1.1447
At4g27652	expressed protein	0.8255
At4g29780	expressed protein	1.4961
At4g35985	senescence/dehydration-associated protein-related	0.6918
At5g12010	expressed protein	0.6498
At5g16830	syntaxin 21 (SYP21)/PEP12 homolog	0.3345

(Time 45) that are known to be universally responsive to abiotic stress.

### Membrane transporters Col-0 time series

**Figure 7:**
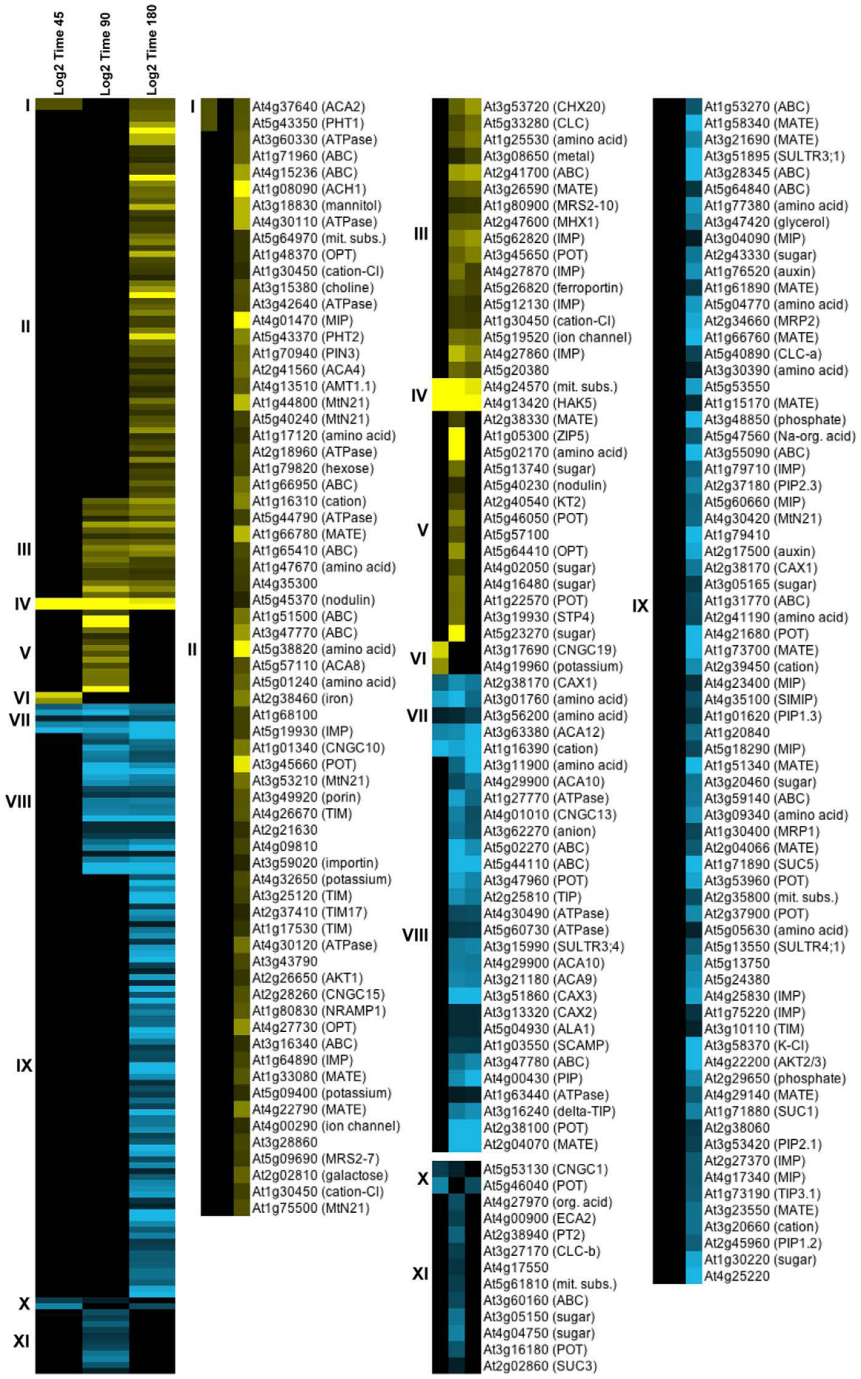
Hierarchical average linkage cluster analysis of transporter gene expression using uncentered correlation. The cluster analysis is based on transporter genes with significant expression at Time 45, 90 or 180. Yellow denotes a higher, and blue a lower expression of a gene in the treated plants versus the control. The figure shows that distinct clusters of expression patterns can be distinguished within the group of transporter genes across the three comparisons. The full cluster set is shown on the left; subsets of the clusters are expanded to the right to allow closer inspection of the differential expression patterns. The first 10 letters of the annotation are provided in the expanded sections. The fully annotated figure can be found in the Supplemental material (Fig. S1).

Of special interest is the differential expression of transporter genes as they may represent a metabolic strategy for tolerance to an elevated magnesium sulfate environment. Over 200 different genes encoding membrane-based transporters were differentially expressed across the Col-0 time series (Table S2). Since the Time 90 and 180 comparisons are not fully controlled for diurnal effects, gene expression differences for several transporter genes of interest to this study were analyzed by Q-PCR using diurnally controlled samples. The number of differentially expressed transporter genes increased from 13 at Time 45, to 74 at Time 90 and 189 at Time 180 (Table 2). The expression of the 217 unique genes encoding transporters across the Col-0 time series was analyzed by cluster algorithms to reveal subsets of genes with corresponding patterns of expression (Fig. 7 and Fig. S1).

**Figure 8:**
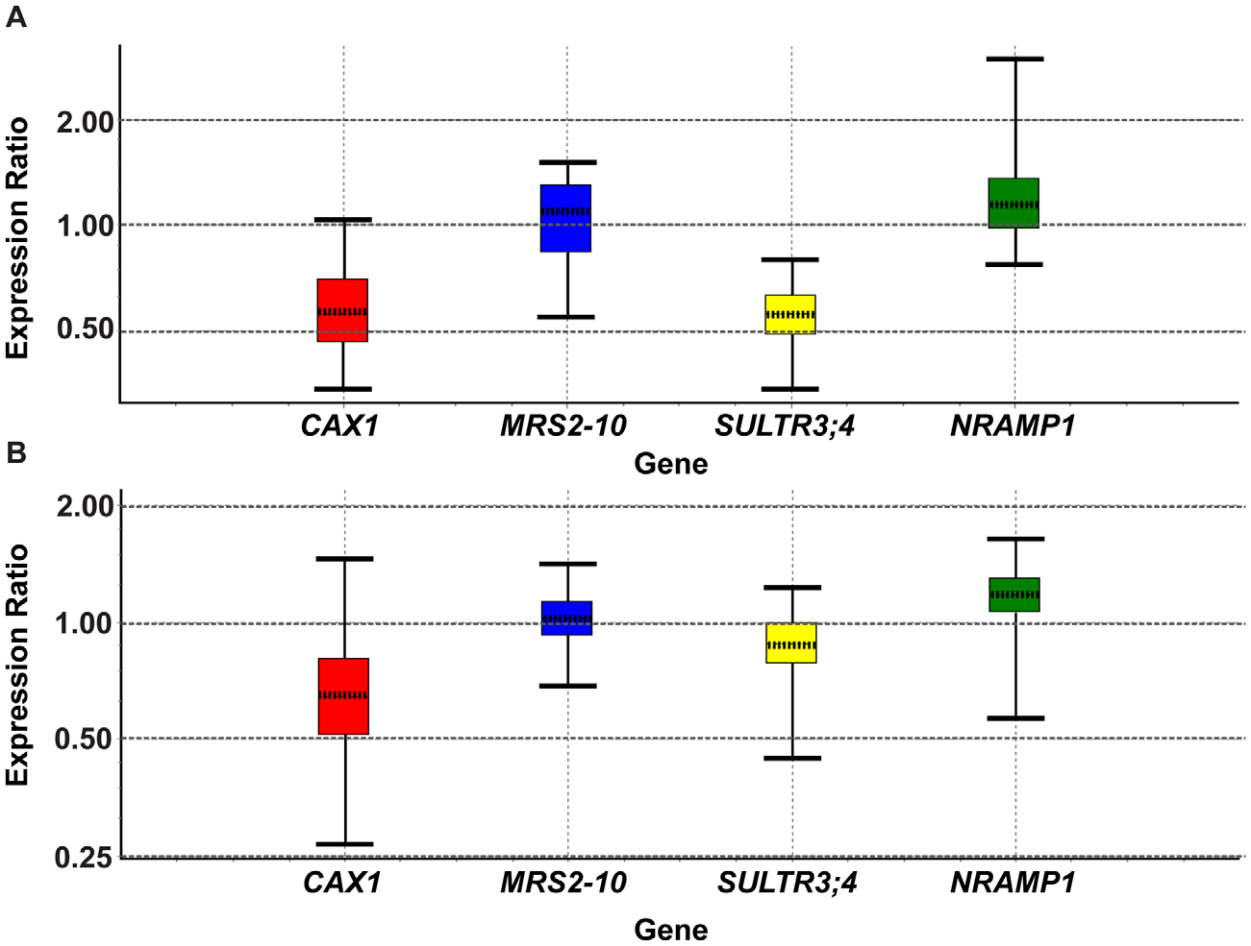
Whisker box plots representing gene expression ratio distributions for the Q-PCR analysis of four transporter genes. The gene expression ratio distributions of transporter genes that showed significant differences in expression in the root transcriptome analysis of Col-0 treated for 180 min. vs Col-0 exposed to a control solution for 45 min are represented by Whisker box plots. Results show permutated expression data that are calculated by the REST 2008 statistical analysis software, which uses randomization techniques. The graphs give an impression of the expression ratio distribution per gene related to the results presented in Table 7. (a) RNA sources were the same as for the transcriptome analysis (Col-0 treated for 180 min. vs Col-0 exposed to a control solution for 45 min.). (b) RNA was extracted from Col-0 treated for 180 min vs Col-0 exposed to a control solution for 180 min. (diurnally controlled samples).

The differential expression of genes encoding known magnesium, sulfate and calcium/proton transporters is summarized in Table 6. The summary shows that the expression of the genes encoding magnesium transporters MRS2-10 and MRS2-7 is slightly up-regulated. Genes encoding sulfate transporters SULTR3;4, SULTR3;1 and SULTR4;1 (a vacuolar H^+^/SO_4_^2−^ cotransporter) show down-regulated expression. The down-regulated expression of the gene encoding SULTR3;4 was confirmed by Q-PCR at 180 min., although its down-regulated expression was less pronounced when controlled for diurnal effects (Table 7, Fig. 8). The gene encoding the vacuolar Mg^2+^/H^+^ antiporter (MHX) shows up-regulated expression, while the vacuolar Ca^2+^/H^+^ antiporters CAX1, CAX2 and CAX3 show down-regulated expression. The down-regulated expression of CAX1 was confirmed by Q-PCR after 180 min. of exposure when controlled for diurnal effects (Table 7, Fig. 8). Besides the genes described above, there were many examples of differentially expressed genes encoding transporters of unknown function belonging to several large transporter gene families. Represented families include the MATE efflux family, the ABC transporter family, the integral membrane family, the major intrinsic protein family, the cation efflux family, the cation-chloride cotransporter family, the anion exchange family, and the ATPase E1–E2 type family. In addition, several genes encoding transporter-related proteins and putative transporters were differentially expressed.

**Table 6:** Genes encoding known magnesium, sulfate and calcium/proton transporters with significant differences in expression in Col-0 at Time 45, 90 and 180.

**Magnesium transporter family:**				
Gene	Description	Log2 Time 45	Log2 Time 90	Log2 Time 180
At1g80900	magnesium transporter (MRS2-10)		0.2182	0.2425
At5g09690	magnesium transporter (MRS2-7)			0.2772
**Sulfate transporter family**				
Gene	Description	Log2 Time 45	Log2 Time 90	Log2 Time 180
At3g51895	sulfate transporter (SULTR3;1)			−2.6591
At5g13550	sulfate transporter (SULTR4;1)			−0.5451
At3g15990	sulfate transporter (SULTR3;4)		−0.7491	−0.8085
**Cation/proton antiporter families**				
Gene	Description	Log2 Time 45	Log2 Time 90	Log2 Time 180
At2g38170	calcium exchanger (CAX1)	−0.5676	−0.8702	−0.7321
At3g13320	calcium exchanger (CAX2)		−0.2668	−0.2707
At3g51860	cation exchanger (CAX3)		−1.1699	−1.1249
At2g47600	magnesium/proton exchanger (MHX1)		0.3887	0.4055
At3g53720	cation/hydrogen exchanger (CHX20)		0.4314	0.6706

**Table 7:** Q-PCR analysis results.

Gene	Type	Relative Expression (Array results)	Std. Error	P-value	Result
Q-PCR results of gene expression in Col-0 treated for 180 min. vs Col-0 exposed to a control solution for 45 min. (RNA sources are the same as for the transcriptome analysis):					
At2g32170	REF	1			
*CAX1*	TRG	0.57 (0.602)	0.436–0.766	0	DOWN
*MRS2-10*	TRG	1.035 (1.183)	0.758–1.377	0.714	
*SULTR3;4*	TRG	0.557 (0.541)	0.462–0.691	0	DOWN
*NRAMP1*	TRG	1.196 (1.317)	0.886–1.515	0.107	
Q-PCR results of gene expression in Col-0 treated for 180 min. vs Col-0 exposed to a control solution for 180 min. (diurnally controlled samples):					
At2g32170	REF	1			
*CAX1*	TRG	0.641 (0.602)	0.429–0.966	0.004	DOWN
*MRS2-10*	TRG	1.017 (1.183)	0.876–1.195	0.763	
*SULTR3;4*	TRG	0.861 (0.541)	0.695–1.059	0.049	DOWN
*NRAMP1*	TRG	1.128 (1.317)	0.970–1.381	0.156	

### Differentially regulated genes *cax1-1*/Col-0 comparison

Only 4 unique transcripts showed a significant difference in expression between the *cax1-1* and Col-0 sample sets treated for 180 minutes (Table 8). Among those, the transcript with the smallest q-values and largest differences in expression was *CAX1*, the gene that was knocked out in the mutant *cax1-1*, and which is represented on the microarray by two different probes. The other three transcripts included At3g01345, an expressed protein with similarity to beta-galactosidases in Arabidopsis, At4g07526, an unknown protein with similarity to other unknown proteins in Arabidopsis, and chromosomal region CHR2:011819877–011819818, which corresponds to a portion of the mitochondrial photorespiration gene At2g27730.

**Table 8:** Transcripts with differential expression at q<0.05 between *Arabidopsis thaliana cax1-1* and Col-0 treated for 180 min.

q-value	Log2(FC)	Gene and DNA region description
1.66E-09	−3.0461	calcium exchanger (*CAX1*) [At2g38170.1]
3.28E-07	−3.8209	calcium exchanger (*CAX1*) [At2g38170.3]
0.001201795	−2.7293	Expressed protein [At3g01345.1]
0.004291231	−1.1998	hypothetical protein [At4g07526.1]
0.005396832	−1.0903	Unknown [CHR2:011819877–011819818]

The table shows that 5 transcripts are identified with a significant difference in expression. Two of the transcripts are identical (At2g38170), which means that 4 unique transcripts show a significant difference in expression between *cax1-1* and Col-0 treated for 180 min.

## Discussion

The use of regolith on Mars with high levels of hydrated sulfate minerals for bioregenerative life support systems will lead to exposure of plant roots to supra-optimal concentrations of both Mg^2+^ and SO_4_^2−^ ions in the soil solution. The results in this study show that knockout mutant lines of the known genes in Arabidopsis encoding root plasma membrane based uptake transporters of Mg^2+^ and SO_4_^2−^ ions failed to confer an adaptive advantage to a MgSO_4_·7H_2_O enriched environment. Because *sel1-10* mutants are known to take up 80% less sulfate than their wildtype background Ws, these results mean that phytotoxic effects of magnesium sulfate on overall plant growth are dominated by the Mg^2+^ cation. Overexpression of *AtMRS2-10* (*AtMGT1*) in *Nicotiana benthamiana* led to increased accumulation of magnesium (Mg), manganese (Mn), and iron (Fe) per unit dry weight and per plant compared to wildtype plants [29]. However, the potential role of AtMRS2-10 as a major uptake transporter of Mg^2+^ in Arabidopsis remains uncertain based on the observation that *mrs2-10* plants did not show a significant difference in leaf Mg levels compared to Col-0 under standard nutrient conditions. This could explain why, despite the indication that Mg dominantly affects overall plant growth, the *mrs2-10* mutant did not show a significant improvement in growth performance compared to Col-0. Interestingly, the dominant role of Mg is also supported by the relative tolerance of the *cax1-1* vacuolar H^+^/Ca^2+^ transport mutant plants to elevated levels of MgSO_4_·7H_2_O. The soil data shown in Figures 5 and 6 are in line with the relative tolerance of *cax1-1* on agar to high concentrations of dissolved MgCl_2_, a mineral in which the Mg^2+^ cation is the dominant phytotoxic factor [16]. In addition, Bradshaw et al (2005) observed that *cax1* mutants are better able to tolerate the low Ca∶Mg ratios characteristic of terrestrial serpentine soils. The CAX1 knockout mutation does not fully eliminate the effects of high magnesium sulfate on plant growth performance; the absolute FW shoot biomass of *cax1-1* grown on soil for four weeks was still low (20%) compared to untreated Col-0.

The primary Arabidopsis Col-0 root transcriptome responses to elevated levels of magnesium sulfate suggest a number of candidate genes that could play a role in enhancing the growth performance of plants exposed to this nutrient stress. Transcriptome responses of Arabidopsis Col-0 roots exposed to MgSO_4_·7H_2_O versus a control solution for 45 min. revealed over 300 differentially expressed genes. Genes of known functional category include those encoding enzymes involved in hormone metabolism, transcription factors, calcium-binding proteins, kinases, disease resistance proteins, cell wall related proteins, and membrane-based transporters. The biological significance of this seemingly diverse set of gene classes illustrates the comprehensive nature of a primary abiotic stress response. The differentially expressed genes at Time 45 encoding enzymes in the ABA, ethylene and jasmonate biosynthesis pathways point to possible changes in the synthesis of these phytohormones. ABA, ethylene, and jasmonic acid have all been implicated in adaptive mechanisms of osmotic stress, ion-mediated signal transduction and the regulation of transporters [30], [31], [32]. The exact role of many of the differentially expressed genes at Time 45 encoding transcription factors is currently unknown, although some are associated with tolerance to other abiotic stresses. The up-regulation at Time 45 of the gene encoding ZAT7 is for example in line with the finding that ZAT7 renders plants more tolerant to salinity stress when constitutively expressed [33]. The down-regulation at Time 45 of the gene encoding WRKY70 correlates with the observation that WRKY70 functions as a negative regulator of developmental senescence and is involved in plant defense signaling pathways [34]. The majority of transcripts encoding kinases that are differentially expressed at Time 45 encode uncharacterized protein kinases as well as receptor-like protein kinases (RLKs) with extracytoplasmic leucine-rich repeats (LRRs) or with an extracellular lectin-like domain. RLKs are membrane-spanning proteins with a predicted signal sequence and a cytoplasmic kinase domain that have been implicated in a wide range of signal transduction pathways [35]. The up-regulated expression of the gene encoding CBL-interacting protein kinase CIPK9 at Time 45 is consonant with the fact that CIPK9 is required for low-potassium tolerance in Arabidopsis [36]. Genes encoding proteins that belong to the transcription factor or kinase categories might be transcriptional or post-translational regulators of transporters [37].

Within the group of genes encoding membrane based transporters, the Ca^2+^/H^+^ antiporter CAX1 is one of the few transporter genes that are already differentially expressed at Time 45. Its down-regulated expression is in line with our finding that the *cax1-1* mutant performed better compared to wildtype when grown on soil with elevated levels of magnesium sulfate. The slightly up-regulated expression of the gene encoding magnesium transporter MRS2-10 at Time 90 and 180 agrees with the observation that a knockout mutant of *AtMRS2-10* (*mrs2-10*) does not show improved growth in the form of higher FW shoot biomass compared to Col-0 at elevated levels of magnesium sulfate. MRS2 transporters have been shown to transport other ions in addition to Mg^2+^ [29], [38]. Schock et al. (2002) speculate that, in line with the large number of *AtMRS2* family members, the function of the *AtMRS2* gene family may be the maintenance of metal ion homeostasis in different cellular compartments (i.e. over different cellular membrane systems). The expression of the gene encoding SULTR1;2, a high-affinity sulfate transporter, showed no significant differences at the three time points. The fact that expression of the gene is not down-regulated in the early adaptation response of Arabidopsis roots to high levels of magnesium sulfate supports the outcome of the FW shoot biomass comparison between the *AtSULTR1;2* knockout mutant *sel1-10* and Col-0, which showed no advantage for *sel1-10*. The gene encoding EIL3, one of the known transcriptional regulators of *SULTR1;2* [39], did not show a significant difference in expression at any of the time points either.

Interestingly, two sulfate transporter genes of the same family as *SULTR1;2* show down-regulated expression; the gene encoding SULTR3;4 at Time 90 and 180, and the gene encoding SULTR3;1 at Time 180. The spatial and subcellular localization of these transporters is not known, and so far no influence on the expression of Group 3 sulfate transporters by the sulfur status of plants has been reported [40]. This study shows for the first time that genes encoding Group 3 sulfate transporters SULTR3;1 and SULTR3;4 are differentially expressed in roots upon exposure to high levels of sulfate. The expression of the gene encoding another sulfate transporter - the tonoplast-localized H^+^/SO_4_^2−^ cotransporter SULTR4;1 - is also down-regulated at Time 180, pointing at the possible retention of excess SO_4_^2−^ in the vacuole. Similarly, the up-regulated expression of the gene encoding the vacuolar MHX transporter indicates the possible storage of excess Mg^2+^ in the vacuole. Both *MHX* and *SULTR1;4* could be seen as marker genes for excess magnesium sulfate recognition by the plant, since vacuolar storage of excess ions is a well-known defense mechanism against ion stress.

Several members of the *CAX* (cation exchanger) family show down-regulated expression in response to elevated magnesium sulfate. The Ca^2+^/H^+^ antiporter CAX1, which shows down-regulated expression across the time points, is localized to the tonoplast and responsible for 50% of the Ca^2+^/H^+^ antiport activity there [16]. CAX2 has been shown to transport Ca^2+^ and Mn^2+^ into the vacuole in Arabidopsis and other plant species [41], and CAX3 is known as a weak Ca^2+^ vacuolar transporter. CAX1 and CAX3 can be combined as “hetero-CAX” complexes to form functional transporters with distinct transport properties [42]. The down-regulated expression of the genes encoding these three vacuolar Ca^2+^/H^+^ antiporters indicates a possible shortage of calcium in the cytosol upon high magnesium sulfate exposure.

In total, over 200 differentially expressed genes encoding membrane-based transporters were identified across the Col-0 time series. Among the genes encoding transporters of known and unknown function, candidates for improving plant tolerance to high magnesium sulfate may be found. Transporters of unknown function include for example major intrinsic proteins, cyclic nucleotide gated channels, integral membrane proteins, cation efflux family proteins, ATPase E1–E2 type family proteins, MATE efflux family proteins, transporter-related proteins and putative transporters.

The differential expression of only four unique transcripts between *cax1-1* and Col-0 plants after 180 min. of MgSO_4_·7H_2_O treatment indicates that the root transcriptome responses are virtually identical for the two genotypes at this time after initiation of treatment. In our transcriptome study we see down-regulation of the gene encoding vacuolar sulfate efflux transporter SULTR4;1 at Time 180, and up-regulation of the gene encoding vacuolar magnesium influx transporter MHX at Time 90 and Time 180 in Col-0 exposed to elevated magnesium sulfate. Both of these responses point to storage of excess magnesium and sulfate ions in the vacuole. This means that the profile is most probably not performed at a time point before plants recognize excess magnesium sulfate, especially since over 3000 differentially expressed genes were detected in Col-0 at Time 180, in addition to the vacuolar marker genes. We could therefore not foresee that after 180 minutes of exposure there were no more than four differentially expressed transcripts between the *cax1-1* mutant and Col-0. One of the main reasons for the limited number of differentially regulated transcripts between the genotypes at this time after initiation of treatment may be that *CAX1* is one of the few transporter genes that are already differentially expressed after 45 min. of exposure in Col-0, which means that down-regulated expression of *CAX1* is an important natural response of Arabidopsis to elevated levels of magnesium sulfate. The response of *CAX1* and other *CAX* family members in the Col-0 time series suggests that a deficiency of calcium caused by high levels of magnesium and possibly by calcium sulfate precipitation could play a major part in the reduction of plant growth performance in the presence of high magnesium sulfate concentrations. The difference between the immediately down-regulated level of CAX1 in Col-0 and the absence of CAX1 in the *cax1-1* knockout mutant is apparently not sufficiently large at 180 min. after initiation of treatment to reveal many of the downstream molecular processes eventually leading to enhanced growth performance and reduced leaf Mg content of *cax1-1* compared to Col-0 after several weeks of growth. This result is significant in that follow-up studies contrasting *cax1-1* with Col-0 can look at profiles after possibly even days of exposure.

### Conclusions and perspectives

It was initially thought that the *mrs2-10* and *sel1-10* mutants would have an advantage over wildtype in elevated magnesium sulfate soils, but this was not the case. However, fresh weight shoot biomass analyses showed that although *mrs2-10* and *sel1-10* mutants were not more successful than wildtype in this soil environment, the *cax1* mutants were. Thus, *cax1* mutants are currently the only confirmed genotype with partial tolerance to the phytotoxic levels of magnesium sulfate expected for the regolith on Mars.

To further characterize the response of Arabidopsis to Mars-like levels of magnesium sulfate a set of experiments was conducted to evaluate patterns of gene expression in wildtype (Col-0) and *cax1* mutants. The 325 genes differentially expressed in Col-0 roots after 45 min. of exposure to high magnesium sulfate can be seen as candidate genes for enhanced tolerance. This set of genes included *CAX1*, 18 genes previously associated with enhanced tolerance to broad ranges of abiotic stresses, and several well-characterized genes known to enhance tolerance to other salts like NaCl. Many of the differentially regulated genes contain promoter sequence motifs that are known to play a role in ABA-mediated regulation, such as ABRE-CE. This abundance suggests that ABA plays a role in regulating the stress response to elevated magnesium sulfate. In addition, a rich pool of candidates is found in the 200+ transporter genes that are differentially expressed in Col-0 across the time points. Many genes in this set encode transporters of unknown function. These unknown transporter genes, along with the four differentially regulated transcripts between *cax1-1* and Col-0, are of particular interest as they encode proteins of unknown function whose roles are now at least implicated.

This study functions as a solid basis for the development of Mars soil-compatible plants by reducing the number of potential candidate genes from tens of thousands to several hundred. Future research efforts could determine whether any of these candidate genes, or their potential regulators, can be confirmed as genomic loci for (crop) plant growth enhancement in the presence of mars-like levels of magnesium sulfate.

## Supporting Information

Table S1 Plant growth performance ANOVA. ANOVA (t-test) results are shown for specific genotype concentration effects in the growth experiments.
(0.01 MB XLS)

Table S2 Root transcriptome ANOVA. An ANOVA analysis of differential expression in the root transcriptome.
(0.71 MB XLS)

Figure S1 The fully annotated Figure 7: hierarchical average linkage cluster analysis of transporter gene expression using uncentered correlation. Hierarchical average linkage cluster analysis of transporter gene expression using uncentered correlation. The cluster analysis is based on transporter genes with significant expression at Time 45, 90 or 180. Yellow denotes a higher, and blue a lower expression of a gene in the treated plants versus the control. The figure shows that distinct clusters of expression patterns can be distinguished within the group of transporter genes across the three comparisons.
(0.54 MB PDF)
