# Supplementary material for: Growth Performance and Root Transcriptome Remodeling of Arabidopsis in Response to Mars-Like Levels of Magnesium Sulfate
Source: PLoS One. 2010 Aug 23;5(8):e12348. doi: 10.1371/journal.pone.0012348 (PMC2925951; doi:10.1371/journal.pone.0012348)
Supplement: Figure S1 — The fully annotated Figure 7: hierarchical average linkage cluster analysis of transporter gene expression using uncentered correlation. Hierarchical average linkage cluster analysis of transporter gene expression using uncentered correlation. The cluster analysis is based on transporter genes with significant expression at Time 45, 90 or 180. Yellow denotes a higher, and blue a lower expression of a gene in the treated plants versus the control. The figure shows that distinct clusters of expression patterns can be distinguished within the group of transporter genes across the three comparisons. (0.54 MB PDF) [file pone.0012348.s003.pdf]

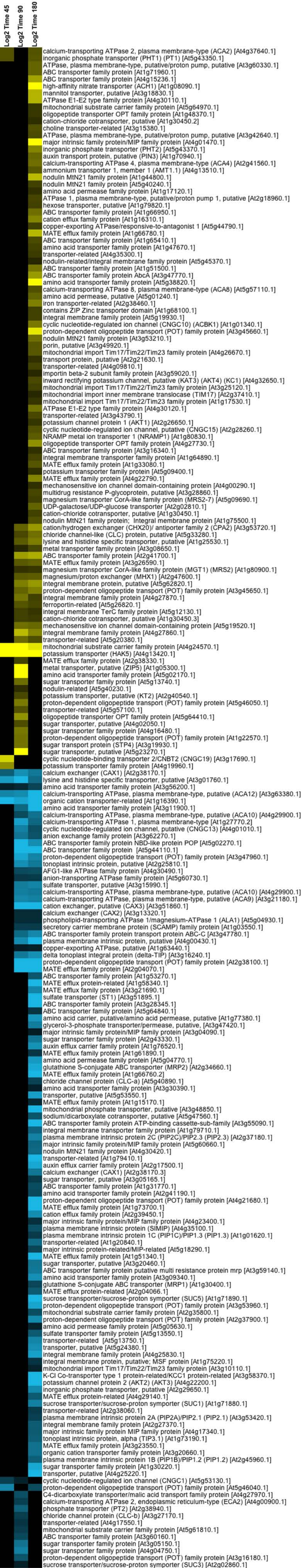
Log2 Time 45
Log2 Time 90
Log2 Time 180
calcium-transporting ATPase 2, plasma membrane-type (ACA2) [At4g37640.1]
inorganic phosphate transporter (PHT1) (PT1) [At5g43350.1]
ATPase, plasma membrane-type, putative/proton pump, putative [At3g60330.1]
ABC transporter family protein [At1g71960.1]
ABC transporter family protein [At4g15236.1]
high-affinity nitrate transporter (ACH1) [At1g08090.1]
mannitol transporter, putative [At3g18830.1]
ATPase E1-E2 type family protein [At4g30110.1]
mitochondrial substrate carrier family protein [At5g64970.1]
oligopeptide transporter OPT family protein [At1g48370.1]
cation-chloride cotransporter, putative [At1g30450.2]
choline transporter-related [At3g15380.1]
ATPase, plasma membrane-type, putative/proton pump, putative [At3g42640.1]
major intrinsic family protein/MIP family protein [At4g01470.1]
inorganic phosphate transporter (PHT2) [At5g43370.1]
auxin transporter protein, putative (PIN3) [At1g70940.1]
calcium-transporting ATPase 4, plasma membrane-type (ACA4) [At2g41560.1]
ammonium transporter 1, member 1 (AMT1.1) [At4g13510.1]
nodulin MtN21 family protein [At1g44800.1]
nodulin MtN21 family protein [At5g40240.1]
amino acid permease family protein [At1g17120.1]
ATPase 1, plasma membrane-type, putative/proton pump 1, putative [At2g18960.1]
hexose transporter, putative [At1g79820.1]
ABC transporter family protein [At1g66950.1]
cation efflux family protein [At1g16310.1]
copper-exporting ATPase/responsive-to-antagonist 1 [At5g44790.1]
MATE efflux family protein [At1g66780.1]
ABC transporter family protein [At1g65410.1]
amino acid transporter family protein [At1g47670.1]
transporter-related [At4g35300.1]
nodulin-related/integral membrane family protein [At5g45370.1]
ABC transporter family protein [At1g51500.1]
ABC transporter family protein AbcA [At3g47770.1]
amino acid transporter family protein [At5g38820.1]
calcium-transporting ATPase 8, plasma membrane-type (ACA8) [At5g57110.1]
amino acid permease, putative [At5g01240.1]
iron transporter-related [At2g38460.1]
contains ZIP Zinc transporter domain [At1g68100.1]
integral membrane family protein [At5g19930.1]
cyclic nucleotide-regulated ion channel (CNGC10) (ACBK1) [At1g01340.1]
proton-dependent oligopeptide transport (POT) family protein [At3g45660.1]
nodulin MtN21 family protein [At3g53210.1]
porin, putative [At3g49920.1]
mitochondrial import Tim17/Tim22/Tim23 family protein [At4g26670.1]
transport protein, putative [At2g21630.1]
transporter-related [At4g09810.1]
importin beta-2 subunit family protein [At3g59020.1]
inward rectifying potassium channel, putative (KAT3) (AKT4) (KC1) [At4g32650.1]
mitochondrial import Tim17/Tim22/Tim23 family protein [At3g25120.1]
mitochondrial import inner membrane translocase (TIM17) [At2g37410.1]
mitochondrial import Tim17/Tim22/Tim23 family protein [At1g17530.1]
ATPase E1-E2 type family protein [At4g30120.1]
transporter-related [At3g43790.1]
potassium channel protein 1 (AKT1) [At2g26650.1]
cyclic nucleotide-regulated ion channel, putative (CNGC15) [At2g28260.1]
NRAMP metal ion transporter 1 (NRAMP1) [At1g80830.1]
oligopeptide transporter OPT family protein [At4g27730.1]
ABC transporter family protein [At3g16340.1]
integral membrane transporter family protein [At1g64890.1]
MATE efflux family protein [At1g33080.1]
potassium transporter family protein [At5g09400.1]
MATE efflux family protein [At4g22790.1]
mechanosensitive ion channel domain-containing protein [At4g00290.1]
multidrug resistance P-glycoprotein, putative [At3g28860.1]
magnesium transporter CorA-like family protein (MRS2-7) [At5g09690.1]
UDP-galactose/UDP-glucose transporter [At2g02810.1]
cation-chloride cotransporter, putative [At1g30450.1]
nodulin MtN21 family protein; Integral membrane protein [At1g75500.1]
cation/hydrogen exchanger (CHX20)/ antiporter family 2 (CPA2) [At3g53720.1]
chloride channel-like (CLC) protein, putative [At5g33280.1]
lysine and histidine specific transporter, putative [At1g25530.1]
metal transporter family protein [At3g08650.1]
ABC transporter family protein [At2g41700.1]
MATE efflux family protein [At3g26590.1]
magnesium transporter CorA-like family protein (MGT1) (MRS2) [At1g80900.1]
magnesium/proton exchanger (MHX1) [At2g47600.1]
integral membrane protein, putative [At5g62820.1]
proton-dependent oligopeptide transport (POT) family protein [At3g45650.1]
integral membrane family protein [At4g27870.1]
ferroportin-related [At5g26820.1]
integral membrane TerC family protein [At5g12130.1]
cation-chloride cotransporter, putative [At1g30450.3]
mechanosensitive ion channel domain-containing protein [At5g19520.1]
integral membrane family protein [At4g27860.1]
transporter-related [At5g20380.1]
mitochondrial substrate carrier family protein [At4g24570.1]
potassium transporter (HAK5) [At4g13420.1]
MATE efflux family protein [At2g38330.1]
metal transporter, putative (ZIP5) [At1g05300.1]
amino acid transporter family protein [At5g02170.1]
sugar transporter family protein [At5g13740.1]
nodulin-related [At5g40230.1]
potassium transporter, putative (KT2) [At2g40540.1]
proton-dependent oligopeptide transport (POT) family protein [At5g46050.1]
transporter-related [At5g57100.1]
oligopeptide transporter OPT family protein [At5g64410.1]
sugar transporter, putative [At4g02050.1]
sugar transporter family protein [At4g16480.1]
proton-dependent oligopeptide transport (POT) family protein [At1g22570.1]
sugar transport protein (STP4) [At3g19930.1]
sugar transporter, putative [At5g23270.1]
cyclic nucleotide-binding transporter 2/CNBT2 (CNGC19) [At3g17690.1]
potassium transporter family protein [At4g19960.1]
cation exchanger (CAX1) [At2g38170.1]
lysine and histidine specific transporter, putative [At3g01760.1]
amino acid transporter family protein [At3g56200.1]
calcium-transporting ATPase, plasma membrane-type, putative (ACA12) [At3g63380.1]
organic cation transporter-related [At1g16390.1]
amino acid transporter family protein [At1g71680.1]
calcium-transporting ATPase, plasma membrane-type, putative (ACA10) [At4g29900.1]
calcium-transporting ATPase 1, plasma membrane-type [At1g27770.2]
cyclic nucleotide-regulated ion channel, putative (CNGC13) [At4g01010.1]
anion exchange family protein [At3g62270.1]
ABC transporter family protein NBD-like protein POP [At5g02270.1]
ABC transporter family protein [At5g44110.1]
proton-dependent oligopeptide transport (POT) family protein [At3g47960.1]
tonoplast intrinsic protein, putative [At2g25810.1]
AFG1-like ATPase family protein [At4g30490.1]
anion-transporting ATPase family protein [At5g60730.1]
sulfate transporter, putative [At3g15990.1]
calcium-transporting ATPase, plasma membrane-type, putative (ACA10) [At4g29900.1]
calcium-transporting ATPase, plasma membrane-type, putative (ACA9) [At3g21180.1]
cation exchanger, putative (CAX3) [At3g51860.1]
calcium exchanger (CAX2) [At3g13320.1]
phospholipid-transporting ATPase 1/magnesium-ATPase 1 (ALA1) [At5g04930.1]
secretory carrier membrane protein (SCAMP) family protein [At1g03550.1]
ABC transporter family protein transport protein ABC-C [At3g47780.1]
plasma membrane intrinsic protein, putative [At4g00430.1]
copper-exporting ATPase, putative [At1g63440.1]
delta tonoplast integral protein (delta-TIP) [At3g16240.1]
proton-dependent oligopeptide transport (POT) family protein [At2g38100.1]
MATE efflux family protein [At2g04070.1]
ABC transporter family protein [At1g53270.1]
MATE efflux protein-related [At1g58340.1]
MATE efflux family protein [At3g21690.1]
sulfate transporter (ST1) [At3g51895.1]
ABC transporter family protein [At3g28345.1]
ABC transporter family protein [At5g64840.1]
amino acid carrier, putative/amino acid permease, putative [At1g77380.1]
glycerol-3-phosphate transporter/permease, putative [At3g47420.1]
major intrinsic family protein/MIP family protein [At3g04090.1]
sugar transporter family protein [At2g43330.1]
auxin efflux carrier family protein [At1g76520.1]
MATE efflux family protein [At1g61890.1]
amino acid permease family protein [At5g04770.1]
glutathione S-conjugate ABC transporter (MRP2) [At2g34660.1]
MATE efflux family protein [At1g66760.2]
chloride channel protein (CLC-a) [At5g40890.1]
amino acid transporter family protein [At3g30390.1]
transporter, putative [At5g53550.1]
MATE efflux family protein [At1g15170.1]
mitochondrial phosphate transporter, putative [At3g48850.1]
sodium/dicarboxylate cotransporter, putative [At5g47560.1]
ABC transporter family protein ATP-binding cassette-sub-family [At3g55090.1]
integral membrane transporter family protein [At1g79710.1]
plasma membrane intrinsic protein 2C (PIP2C)/PIP2.3 (PIP2.3) [At2g37180.1]
major intrinsic family protein/MIP family protein [At5g60660.1]
nodulin MtN21 family protein [At4g30420.1]
transporter-related [At1g79410.1]
auxin efflux carrier family protein [At2g17500.1]
calcium exchanger (CAX1) [At2g38170.3]
sugar transporter, putative [At3g05165.1]
ABC transporter family protein [At1g31770.1]
amino acid transporter family protein [At2g41190.1]
proton-dependent oligopeptide transport (POT) family protein [At4g21680.1]
MATE efflux family protein [At1g73700.1]
cation efflux family protein [At2g39450.1]
major intrinsic family protein/MIP family protein [At4g23400.1]
plasma membrane intrinsic protein (SIMIP) [At4g35100.1]
plasma membrane intrinsic protein 1C (PIP1C)/PIP1.3 (PIP1.3) [At1g01620.1]
transporter-related [At1g20840.1]
major intrinsic protein-related/MIP-related [At5g18290.1]
MATE efflux family protein [At1g51340.1]
sugar transporter, putative [At3g20460.1]
ABC transporter family protein putative multi resistance protein mrp [At3g59140.1]
amino acid transporter family protein [At3g09340.1]
glutathione S-conjugate ABC transporter (MRP1) [At1g30400.1]
MATE efflux protein-related [At2g04066.1]
sucrose transporter/sucrose-proton symporter (SUC5) [At1g71890.1]
proton-dependent oligopeptide transport (POT) family protein [At3g53960.1]
mitochondrial substrate carrier family protein [At2g35800.1]
proton-dependent oligopeptide transport (POT) family protein [At2g37900.1]
amino acid permease family protein [At5g05630.1]
sulfate transporter family protein [At5g13550.1]
transporter-related [At5g13750.1]
transporter, putative [At5g24380.1]
integral membrane protein [At4g25830.1]
integral membrane protein, putative; MSF protein [At1g75220.1]
mitochondrial import Tim17/Tim22/Tim23 family protein [At3g10110.1]
K-Cl Co-transporter type 1 protein-related/KCC1 protein-related [At3g58370.1]
potassium channel protein 2 (AKT2) (AKT3) [At4g22200.1]
inorganic phosphate transporter, putative [At2g29650.1]
MATE efflux protein-related [At4g29140.1]
sucrose transporter/sucrose-proton symporter (SUC1) [At1g71880.1]
transporter-related [At2g38060.1]
plasma membrane intrinsic protein 2A (PIP2A)/PIP2.1 (PIP2.1) [At3g53420.1]
integral membrane family protein [At2g27370.1]
major intrinsic family protein MIP family protein [At4g17340.1]
tonoplast intrinsic protein, alpha (TIP3.1) [At1g73190.1]
MATE efflux family protein [At3g23550.1]
organic cation transporter family protein [At3g20660.1]
plasma membrane intrinsic protein 1B (PIP1B)/PIP1.2 (PIP1.2) [At2g45960.1]
sugar transporter family protein [At1g30220.1]
transporter, putative [At4g25220.1]
cyclic nucleotide-regulated ion channel (CNGC1) [At5g53130.1]
proton-dependent oligopeptide transport (POT) family protein [At5g46040.1]
C4-dicarboxylate transporter/malic acid transport family protein [At4g27970.1]
calcium-transporting ATPase 2, endoplasmic reticulum-type (ECA2) [At4g00900.1]
phosphate transporter (PT2) [At2g38940.1]
chloride channel protein (CLC-b) [At3g27170.1]
transporter-related [At4g17550.1]
mitochondrial substrate carrier family protein [At5g61810.1]
ABC transporter family protein [At3g60160.1]
sugar transporter family protein [At3g05150.1]
sugar transporter family protein [At4g04750.1]
proton-dependent oligopeptide transport (POT) family protein [At3g16180.1]
sucrose transporter/sucrose-proton symporter (SUC3) [At2g02860.1]
